# Indole decreases the virulence of the bivalve model pathogens *Vibrio tasmaniensis* LGP32 and *Vibrio crassostreae* J2-9

**DOI:** 10.1038/s41598-022-09799-1

**Published:** 2022-04-06

**Authors:** Shanshan Zhang, Qian Yang, Songzhe Fu, Colin R. Janssen, Mieke Eggermont, Tom Defoirdt

**Affiliations:** 1 Center for Microbial Ecology and Technology (CMET), Ghent University, Coupure Links 653, 9000 Gent, Belgium; 2 College of Marine Science and Environment, Dalian Ocean University, Dalian, China; 3 Key Laboratory of Environment Controlled Aquaculture (KLECA), Ministry of Education, Dalian, 116023 China; 4 Environmental Toxicology Unit – GhEnToxLab, Ghent University, Coupure Links 653, 9000 Gent, Belgium

**Keywords:** Target identification, Microbiology techniques, Bacteria

## Abstract

Indole signaling plays an important role in bacterial pathogenesis. In this study, the impact of indole on biofilm formation, swimming and swarming motility were explored in *Vibrio tasmaniensis* LGP32 and *Vibrio crassostreae* J2-9, two model pathogens of bivalves. The results showed that indole decreased swimming and swarming motility in both strains, and decreased biofilm formation in *V. crassostreae* J2-9. Furthermore, indole affected a large number of genes at RNA level, including genes related to metabolism, ABC transporters, flagellar assembly, chemotaxis, and response regulators. Finally, the bacterial virulence towards mussel larvae was decreased by pretreatment with indole in both *V. tasmaniensis* LGP32 and *V. crassostreae* J2-9. After 5 days, the survival rate of mussel larvae increased 2.4-fold and 2.8-fold in mussel larvae challenged with *V. tasmaniensis* LGP32 pretreated with 200 µM and 500 µM indole, respectively. The survival rate of mussel larvae increased 1.5-fold and 1.9-fold in mussel larvae challenged with *V. crassostreae* J2-9 pretreated with 200 µM and 500 µM indole, respectively. These data indicate that indole has a significant impact on the virulence of *V. tasmaniensis* LGP32 and *V. crassostreae* J2-9, and indole signaling could be a promising target for antivirulence therapy.

## Introduction

World mussel aquaculture production has been increasing steadily since the 1950s^1^, and currently 94% of the world mussel production comes from aquaculture^2^. In Europe, mussels are economically the most important aquaculture species as its production represents more than one-third of EU aquaculture production. The blue mussel (*Mytilus edulis*) is one of the main cultivated mussel species^3^, and it is known as a robust bivalve species. However, its larviculture appears to be highly susceptible to diseases and mass mortality occurs in dense larval cultures, which is a bottleneck for sustainable development of mussel culture^4^. These mortality events are most probably caused by bacterial pathogens such as vibrios^5,6^. Vibrios belonging to the *Splendidus* clade (i.e. *Vibrio splendidus* and closely related species) can infect a broad host range of aquaculture animals, including mussels^7^, oysters^8^, shrimp^9^, sea cucumber *Apostichopus japonicus*^10^ and fish^11^, resulting in various diseases and high mortality and leading to high economic losses. *Vibrio tasmaniensis* LGP32 and *Vibrio crassostreae* J2-9 are two model bacterial pathogens of marine bivalves such as oyster and mussel^12–15^. It has been reported that *V. tasmaniensis* LGP32 and *V. crassostreae* J2-9 caused more than 80% and 70% mussel larvae mortality respectively after 5 days of challenge^7,15^.

Antibiotics often are the only effective agents that farmers have to protect their animals from bacterial infections. As a result of the frequent use of antibiotics in aquaculture, the adverse multifactorial effects of antibiotic resistance have increasingly been demonstrated and have become a serious problem for public health^16^. Therefore, there is an urgent need for a new strategy replacing the use of antibiotics^17^. Among the newly developed therapeutic strategies, antivirulence therapy has been proposed as a promising alternative. Instead of killing pathogens, antivirulence therapy aims at disarming pathogens in order to inhibit their virulence, thereby preventing them from attacking the host^18^. Amongst the most intensively studied targets for antivirulence therapy are bacterial cell-to-cell communication systems (quorum sensing)^19^. Vibrios typically contain a multichannel quorum sensing system that combines up to three different types of signal molecules that jointly regulate the expression of several genes^20^. However, in contrast to other *Vibrio* species, the multichannel quorum sensing systems were recently shown not to control the virulence of *Vibrio tasmaniensis* and *Vibrio crassotreae*^15^. In recent years, another signaling molecule, indole, has been proposed as potential target for antivirulence therapy against antibiotic-resistant pathogens without affecting their growth^21,22^. Indole is an aromatic signaling molecule that is widespread in the natural environment^23^. It is synthesized from tryptophan by the tryptophanase enzyme (TnaA) in a large number of bacteria including Gram-negative and Gram-positive species, both pathogenic and nonpathogenic^23,24^. As an intercellular, interspecies, and interkingdom signaling molecule, indole plays important roles in bacterial pathogenesis. For example, indole has been reported to control various virulence-related phenotypes (most notably biofilm formation and motility) in various bacterial pathogens, including vibrios such as *Vibrio anguillarum*, *Vibrio campbellii*, *Vibrio cholerae*, *Vibrio harveyi* and *Vibrio parahaemolyticus*^25–28^. In this study, we aimed at investigating the impact of indole on the virulence of the bivalve model pathogens *V. tasmaniensis* LGP32 and *V. crassostreae* J2-9.

## Results

### Quantification of indole produced by *V. tasmaniensis* LGP32 and *V. crassostreae* J2-9

**Figure 1:**
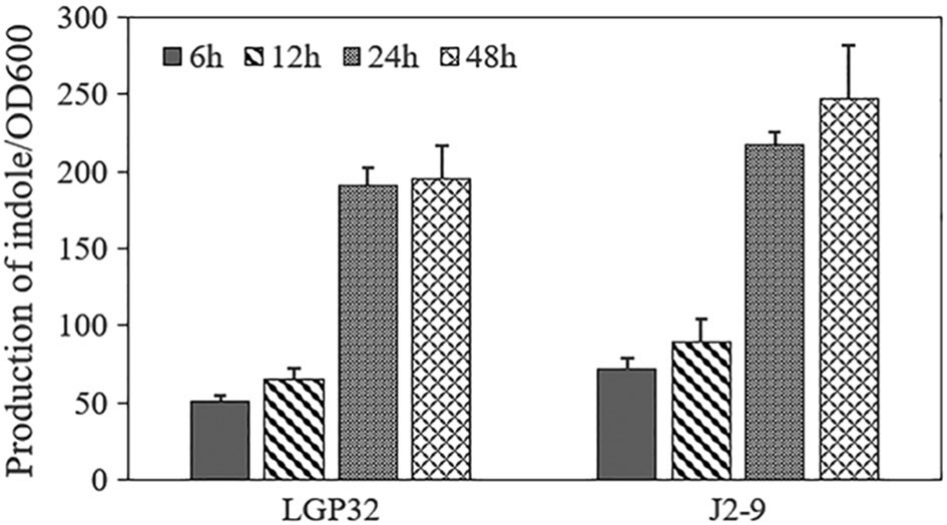
Indole production by *V. tasmaniensis* LGP32 and *V. crassostreae* J2-9. Bacteria were grown in LB_35_ broth and the production of indole was measured using Kovac's reagent. The error bars represent the standard deviation of three independent experiments.

The concentration of extracellular indole produced by *V. tasmaniensis* LGP32 and *V. crassostreae* J2-9 varied as the cell density (OD_600_) changed (Fig. 1). During the first 12 h (exponential phase), *V. tasmaniensis* LGP32 and *V. crassostreae* J2-9 did not produce much indole. After 24 h (early stationary phase), the concentrations of indole reached the highest level around 200 µM in *V. tasmaniensis* LGP32 cultures and 250 µM in *V. crassostreae* J2-9 cultures. The concentration of indole subsequently remained constant until the last sampling point at 48 h (stationary phase).

### Impact of exogenous indole on the growth of *V. tasmaniensis* LGP32 and *V. crassostreae* J2-9

**Figure 2:**
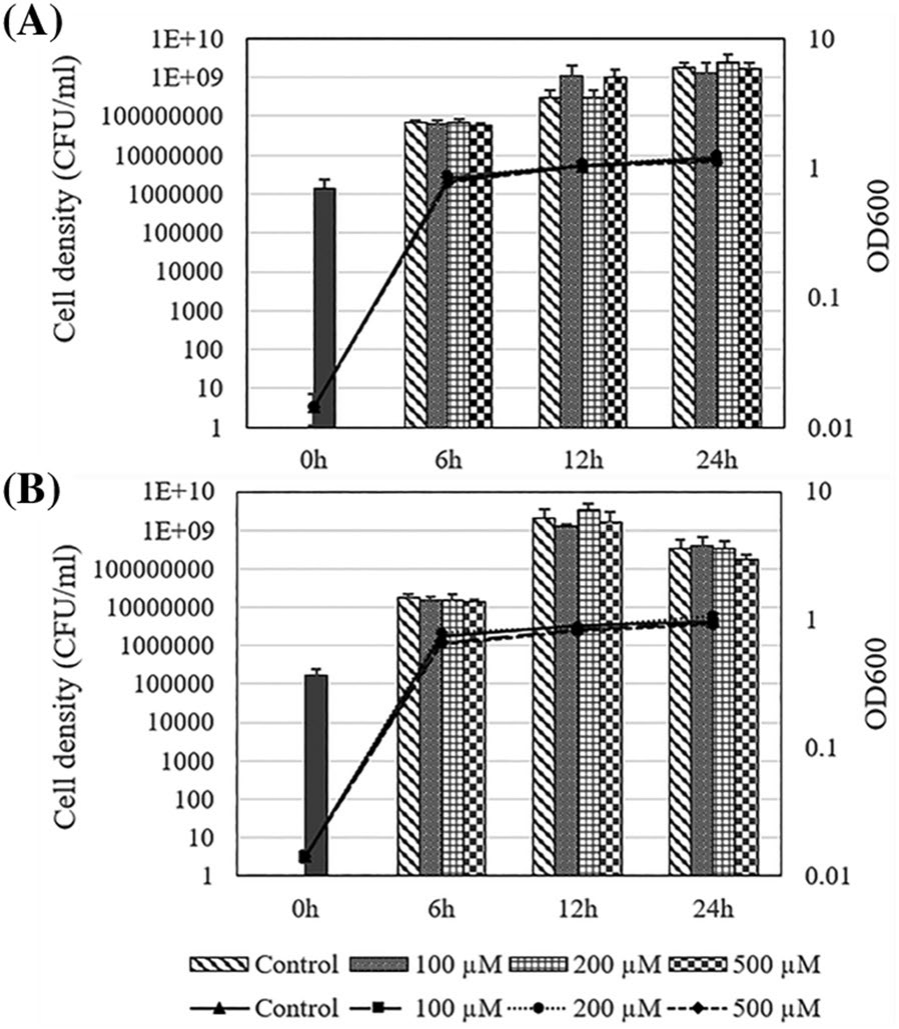
Growth of *V. tasmaniensis* LGP32 (**A**) and *V. crassostreae* J2-9 (**B**) in LB_35_ medium supplemented with various concentrations of indole by measuring OD600 (lines) and plate counting on LB_35_ agar (bars). The error bars represent the standard deviation of three independent experiments.

The growth of *V. tasmaniensis* LGP32 and *V. crassostreae* J2-9 was determined in LB_35_ media supplemented with indole at concentrations of 0, 100, 200 and 500 µM. There were no differences in the growth curves between indole-supplemented cultures and control cultures without indole (Fig. 2).

### Impact of indole on swimming and swarming motility, and biofilm formation

**Figure 3:**
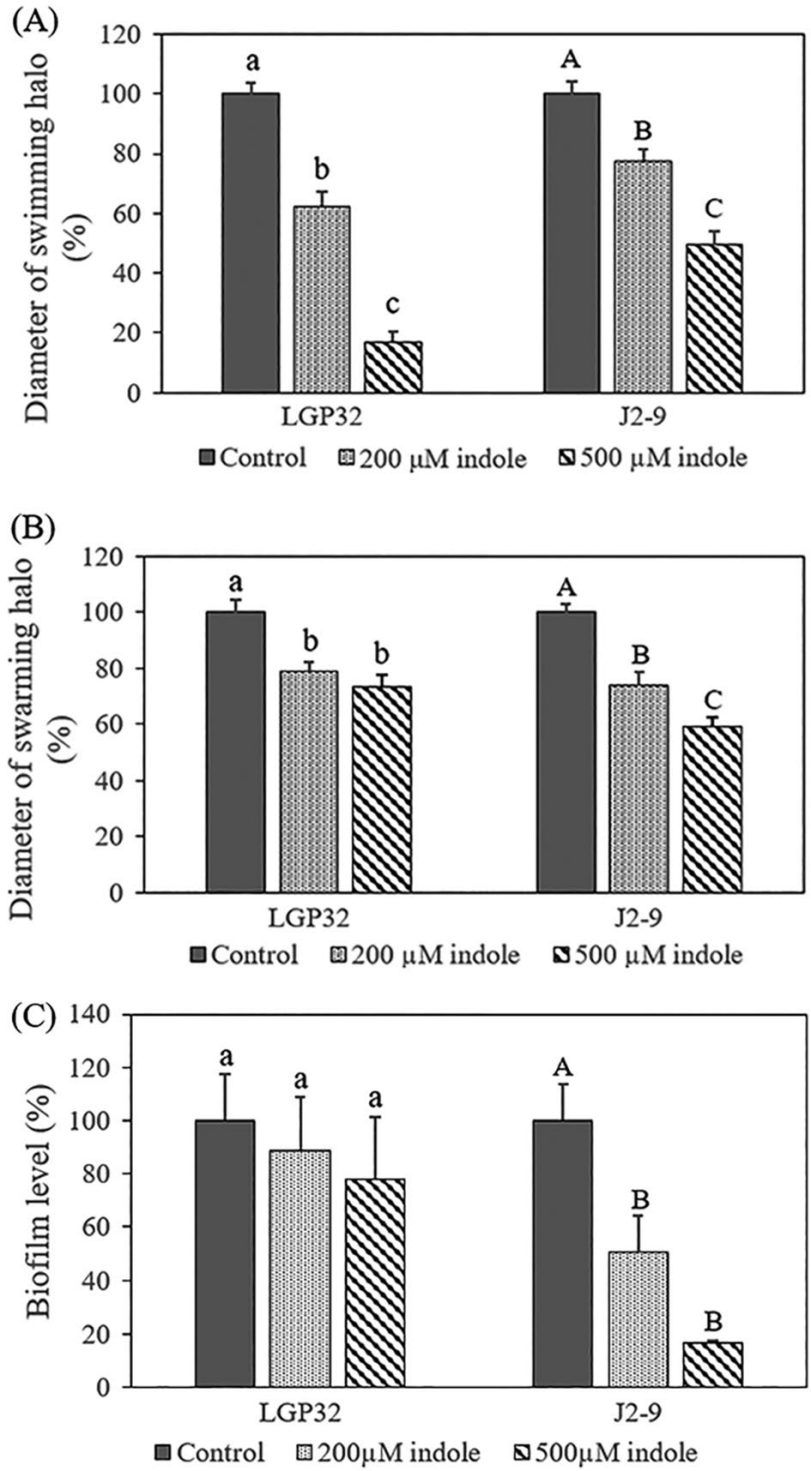
Impact of indole on (**A**) swimming motility, (**B**) swarming motility and (**C**) biofilm formation on polystyrene 96-well plates of *V. tasmaniensis* LGP32 and *V. crassostreae* J2-9. Data are presented as the mean ± SD of six replicates for swimming and swarming motility and three independent experiments for biofilm formation. For each strain, the swimming motility, swarming motility and biofilm formation in the control treatment was set at 100% and the other treatments were normalized accordingly. Different letters indicate significant differences (One way ANOVA with Tukey’s post hoc test; P < 0.01).

In order to determine the mechanism of how indole affects the virulence of *V. tasmaniensis* LGP32 and *V. crassostreae* J2-9, we investigated its impact on swimming and swarming motility and biofilm formation. First, we tested the impact of indole on swimming motility. The results showed that indole inhibited the swimming motility of both *V. tasmaniensis* LGP32 and *V. crassostreae* J2-9 (Fig. 3A). After 2 days, the diameters of the swimming halos produced by cultures of *V. tasmaniensis* LGP32 and *V. crassostreae* J2-9 treated with 500 µM of indole were decreased to 17% and 49%, respectively, of those of untreated cultures. In the second experiment, swarming motility exhibited by *V. tasmaniensis* LGP32 and *V. crassostreae* J2-9 on 0.8% agar was also significantly reduced by indole (Fig. 3B). After 7 days, the swarming motility halos produced by *V. tasmaniensis* LGP32 were 21% and 27% smaller in the presence of 200 and 500 µM indole, respectively, when compared to untreated cultures. The swarming motility of *V. crassostreae* treated with 200 and 500 µM of indole was 26% and 41% smaller, respectively, than the motility of untreated cultures. In the third experiment, we determined the impact of indole on biofilm formation. Indole was found to significantly decrease the biofilm formation in *V. crassostreae* J2-9 but showed no effect on biofilm formation of *V. tasmaniensis* LGP32 (Fig. 3C). The biofilm in *V. crassostreae* J2-9 was reduced to 49% and 17% in the presence of 200 and 500 µM indole, respectively, compared with that of the non-treated control.

### Transcriptional profiling of *V. tasmaniensis* LGP32 during indole treatment

**Figure 4:**
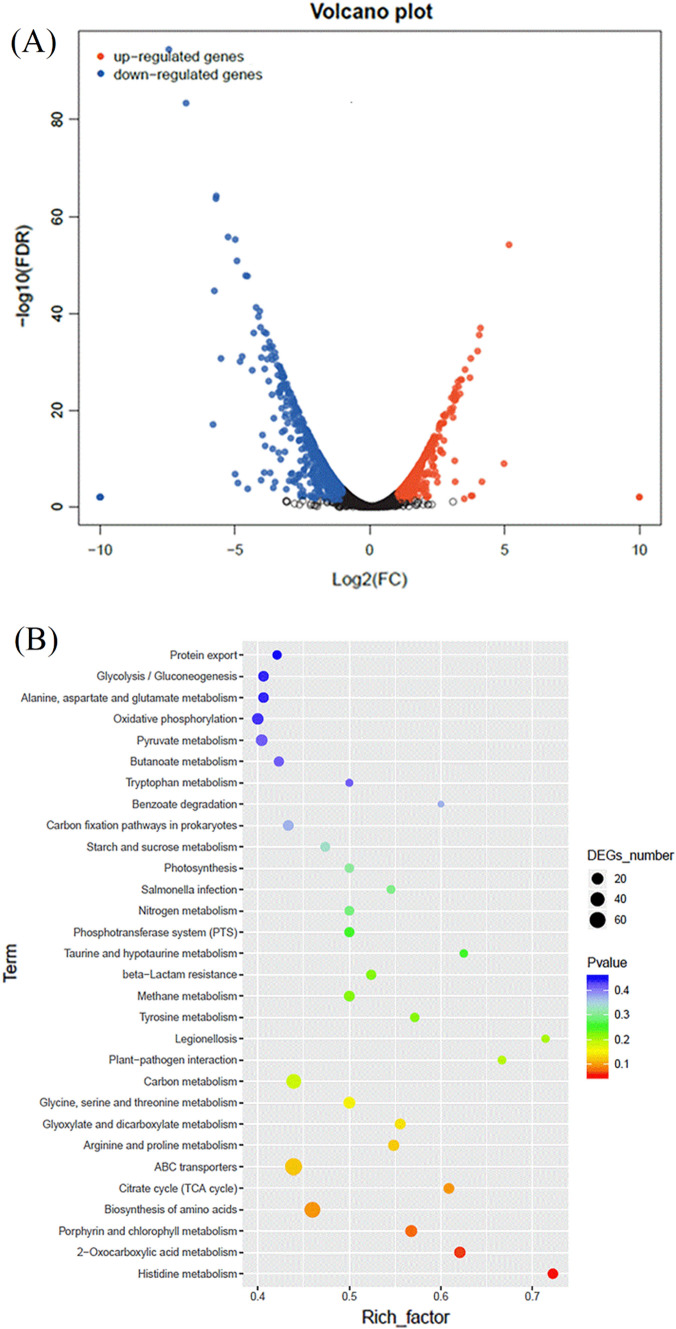
The difference between *V. tasmaniensis* LGP32 without and with 500 µM indole at the whole transcriptome level. (**A**) Volcano plot of differentially expressed genes (DEGs). Red indicates upregulated expression, blue indicates downregulated expression and black indicates no significantly differential expression. FC: fold change; FDR: false discovery rate. (**B**) Functional enrichment of differentially expressed genes on KEGG categorization in *V. tasmaniensis* LGP32 (control vs. 500 µM indole). The Rich factor is the ratio of differentially expressed gene numbers annotated in this pathway term to all gene numbers annotated in this pathway term. The higher the Rich factor, the higher the degree of pathway enrichment. The q value is the corrected p value (q = p*length(p)/rank(p)); a lower value indicates higher pathway enrichment.

Since the above results indicated that indole inhibits the production of several virulence factors, we sought to further determine the genes that are transcriptionally regulated in this context. Thus, we performed comparative RNA sequencing (RNA-seq) on strain LGP32 (whose genome is publicly available on NCBI) during growth in the presence or absence of 500 µM indole. The results identified 697 genes displaying different expression levels, of which 269 were up-regulated while 428 were down-regulated (Fig. 4). The Kyoto Encyclopedia of Genes and Genomes (KEGG) enrichment analysis revealed that a large number of differentially expressed genes (DEGs) were related to metabolism, ABC transporters, flagellar assembly, chemotaxis, and response regulators (Table 1).

**Table 1:** Responsive genes up-regulated and down-regulated in *Vibrio tasmaniensis* LGP32 during treatment with 500 µM indole.

Cluster	Gene	logFC	Type	pvalue	Function
ABC transporter permease	VS_RS18475	2.055973559	Up	5.06E−12	Putative transmembrane transport protein
	VS_RS18535	2.112345721	Up	1.00E−12	Efflux RND transporter permease subunit
	VS_RS18540	2.018513298	Up	1.10E−08	Efflux RND transporter periplasmic adaptor subunit
	VS_RS18545	2.343129132	Up	6.02E−15	Efflux RND transporter periplasmic adaptor subunit
	VS_RS18795	2.21694386	Up	1.21E−14	DHA2 family efflux MFS transporter permease subunit
	VS_RS19200	2.817138312	Up	5.86E−21	ABC transporter permease
	VS_RS20695	4.980248019	Up	9.80E−11	ABC transporter permease
	VS_RS12520	2.561732455	Up	3.65E−18	Iron ABC transporter permease
ATP synthase	VS_RS19080	2.284974235	Up	4.00E−11	F0F1 ATP synthase subunit A
	VS_RS19085	4.160615584	Up	1.06E−06	Putative ATP synthase subunit C
	VS_RS19090	1.972216459	Up	7.91E−05	F0F1 ATP synthase subunit B
	VS_RS19095	2.430108424	Up	5.26E−09	MULTISPECIES: ATP synthase subunit delta 2
	VS_RS19100	2.336035166	Up	2.08E−13	MULTISPECIES: F0F1 ATP synthase subunit alpha
	VS_RS19105	2.042150854	Up	3.21E−08	MULTISPECIES: F0F1 ATP synthase subunit gamma
	VS_RS19110	1.790491474	Up	4.08E−08	F0F1 ATP synthase subunit beta
	VS_RS19195	2.347242323	Up	1.64E−15	Microcin C ABC transporter ATP-binding protein YejF
Glycogen metabolism	VS_RS15610	1.596031056	Up	1.07E−06	Pullulanase-type alpha-1,6-glucosidase
	VS_RS15675	2.636153819	Up	1.48E−19	MULTISPECIES: glycogen/starch/alpha-glucan phosphorylase
	VS_RS15680	2.092543897	Up	1.09E−12	4-Alpha-glucanotransferase
Flagella	VS_RS03755	− 2.079204877	Down	6.12E−15	Polar flagellin B
	VS_RS03760	− 2.222746448	Down	9.71E−17	Flagellin
	VS_RS03765	− 1.59963616	Down	4.23E−08	Flagellar protein FlaG
Stress	VS_RS00405	− 3.330418851	Down	8.25E−32	Universal stress protein A
	VS_RS00415	− 1.523398156	Down	5.46E−09	MULTISPECIES: universal stress protein B
	VS_RS07555	− 3.29755626	Down	1.12E−27	MULTISPECIES: universal stress protein A
Glycerol metabolism	VS_RS15345	− 2.643136518	Down	3.52E−22	Glycerol-3-phosphate dehydrogenase
	VS_RS15355	− 4.989217537	Down	8.10E−59	Glycerol kinase
	VS_RS21245	− 3.165892799	Down	4.78E−27	MULTISPECIES: sn-glycerol-3-phosphate ABC transporter ATP-binding protein UgpC
	VS_RS07525	− 2.58950755	Down	1.19E−20	Glycerol-3-phosphate transporter
Chemotaxis	VS_RS15830	− 2.1220644	Down	1.73E−15	Methyl-accepting chemotaxis protein
	VS_RS19890	− 2.589006414	Down	9.30E−20	Methyl-accepting chemotaxis protein
	VS_RS20855	− 3.088890925	Down	3.23E−24	MULTISPECIES: methyl-accepting chemotaxis protein
	VS_RS20915	− 3.323245847	Down	3.23E−27	Methyl-accepting chemotaxis protein
	VS_RS01160	− 2.795950233	Down	1.32E−23	Methyl-accepting chemotaxis protein
	VS_RS01930	− 2.235053394	Down	5.20E−16	Methyl-accepting chemotaxis protein
	VS_RS08445	− 2.712718428	Down	2.45E−22	Methyl-accepting chemotaxis protein
Response regulators	VS_RS18640	− 2.88617186	Down	8.86E−22	MULTISPECIES: XRE family transcriptional regulator
	VS_RS19805	− 3.155331292	Down	5.76E−18	Two-component system response regulator
	VS_RS21020	− 3.264893109	Down	8.73E−23	Sigma-54-dependent Fis family transcriptional regulator
	VS_RS21270	− 3.681928235	Down	1.11E−35	MULTISPECIES: LacI family DNA-binding transcriptional regulator
	VS_RS01820	− 3.890204996	Down	8.43E−36	DeoR family transcriptional regulator
	VS_RS02450	− 4.992855982	Down	2.09E−08	MULTISPECIES: ArsR family transcriptional regulator
	VS_RS04480	− 2.48262716	Down	4.77E−16	Response regulator
	VS_RS05355	− 2.162622235	Down	1.91E−08	LysR family transcriptional regulator
	VS_RS07145	− 2.20970694	Down	4.67E−16	Snal regulator
	VS_RS08545	− 2.790069971	Down	1.72E−20	Response regulator

*FC* fold change.

Based on the transcriptomic analysis, several genes associated with flagellar motility were found to be downregulated in *V. tasmaniensis* LGP32 that was treated with indole, including genes encoding flagellin, polar flagellin B, and flagellar protein FlaG. Genes associated with methyl-accepting chemotaxis proteins, universal stress protein A, and carbon starvation protein A were also downregulated, indicating that the treatment with indole significantly decreased the expression of genes related to chemotaxis and stress response. Notably, one gene encoding a two-component system response regulator was also downregulated. Further, the expression of several flagellin related genes including *flaB* (encoding flagellin), *flaE* (encoding polar flagellin), and *flaN* (encoding polar flagellin B) were decreased, although the changes were not significant (fold change between 1.0 and 1.5; Table S1). In contrast, a substantial number of genes related to ABC transporter permease and ATP synthase were significantly upregulated (Table 1).

### Impact of indole on the expression of selected genes in *V. tasmaniensis* LGP32 and *V. crassostreae* J2-9

**Figure 5:**
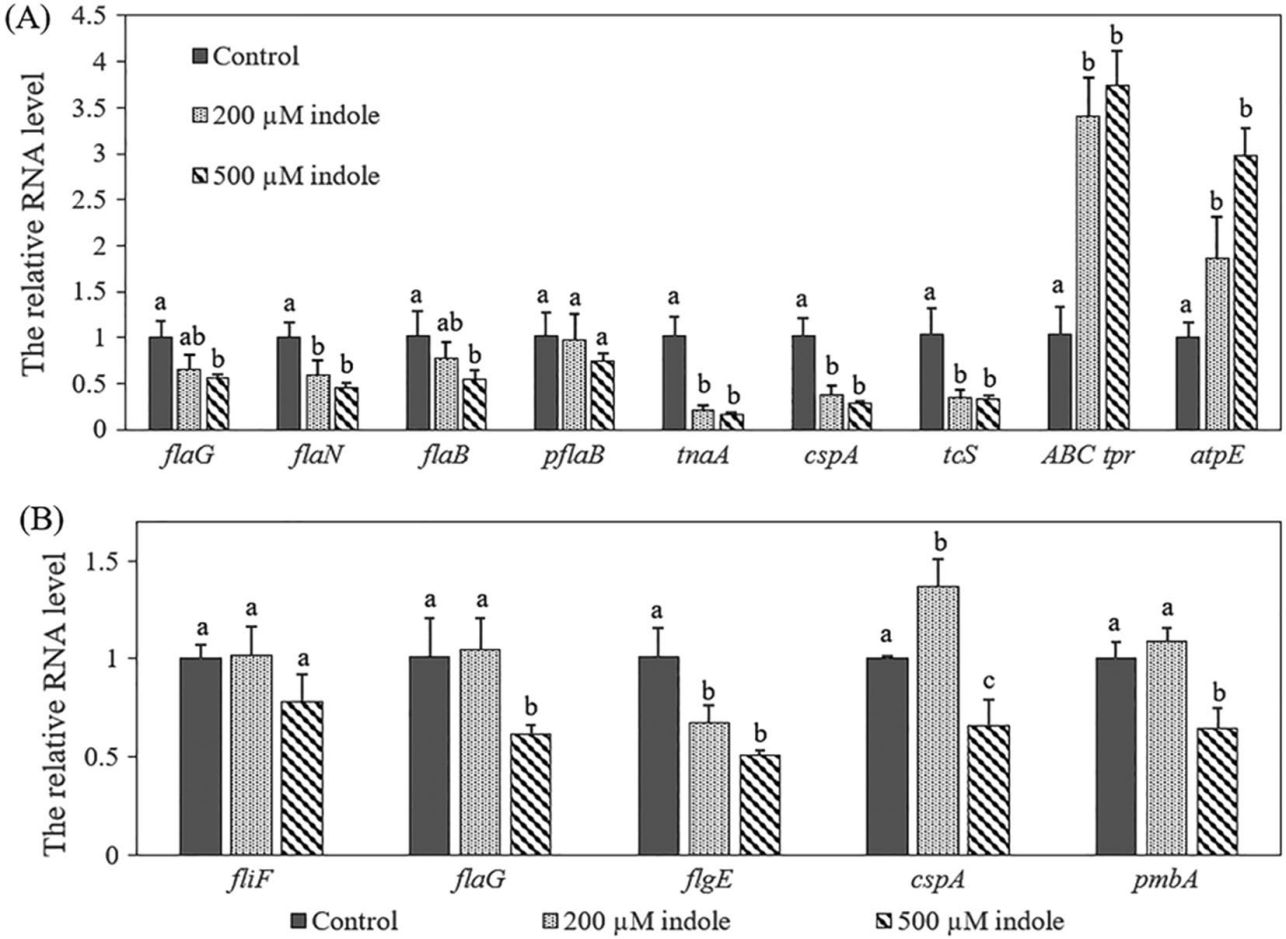
The impacts of indole on mRNA levels of selected genes in *V. tasmaniensis* LGP32 (**A**) and *V. crassostreae* J2-9 (**B**). Bacteria were grown in LB_35_ medium supplemented with indole at 0, 200 or 500 µM and cultured for 6 h, respectively. Cells were collected for total RNA extraction and used for real-time RT-PCR. The relative mRNA levels were normalized to that of 16S rRNA. The mRNA level of the treatment without indole was set at 1 and the other treatments were normalized accordingly using the ΔΔC_T_ method. Error bars represent the standard deviation of three independent experiments. Different letters indicate significant differences (One way ANOVA with Tukey’s post hoc test; P < 0.01).

To confirm the transcriptomic analysis in *V. tasmaniensis* LGP32, we further validated the results by RT-qPCR. The expression of seven genes were verified in *V. tasmaniensis* LGP32 treated with 200 and 500 µM indole, i.e. *flaG* (encoding flagellar protein), *flaN* (encoding flagellin), *flaB* (encoding polar flagellin), *pflaB* (encoding polar flagellin B), *tnaA* (encoding tryptophanase, which produces indole), *cspA* (encoding carbon starvation protein A) and *tcS* (encoding two-component system response regulator) (Fig. 5A). Consistent with the transcriptomic results, all of these genes were down-regulated (p < 0.01). qPCR also confirmed that genes associated with ABC transporter permease (*ABC tpr* encoding ABC transporter permease-related gene), ATP synthase (*atpE* encoding putative ATP synthase subunit C) were upregulated.

To further investigate the impact of indole on *V. crassostreae* J2-9, the genes *fliF* (encoding flagellar M-ring protein), *flaG* (encoding flagellar protein), flgE (encoding flagellar hook protein), *cspA* (encoding carbon starvation protein A) and *pmbA* (encoding metalloprotease PmbA) were tested by RT-qPCR. As shown in Fig. 5B, 500 µM indole also down-regulated the mRNA levels of genes related to motility including *fliF*, *flaG*, and *flgE*. These data were substantiated by the fact that indole could significantly inhibit the swimming and swarming motility of *V. crassostreae* J2-9.

### Impact of indole on the virulence of vibrios towards mussel larvae

**Figure 6:**
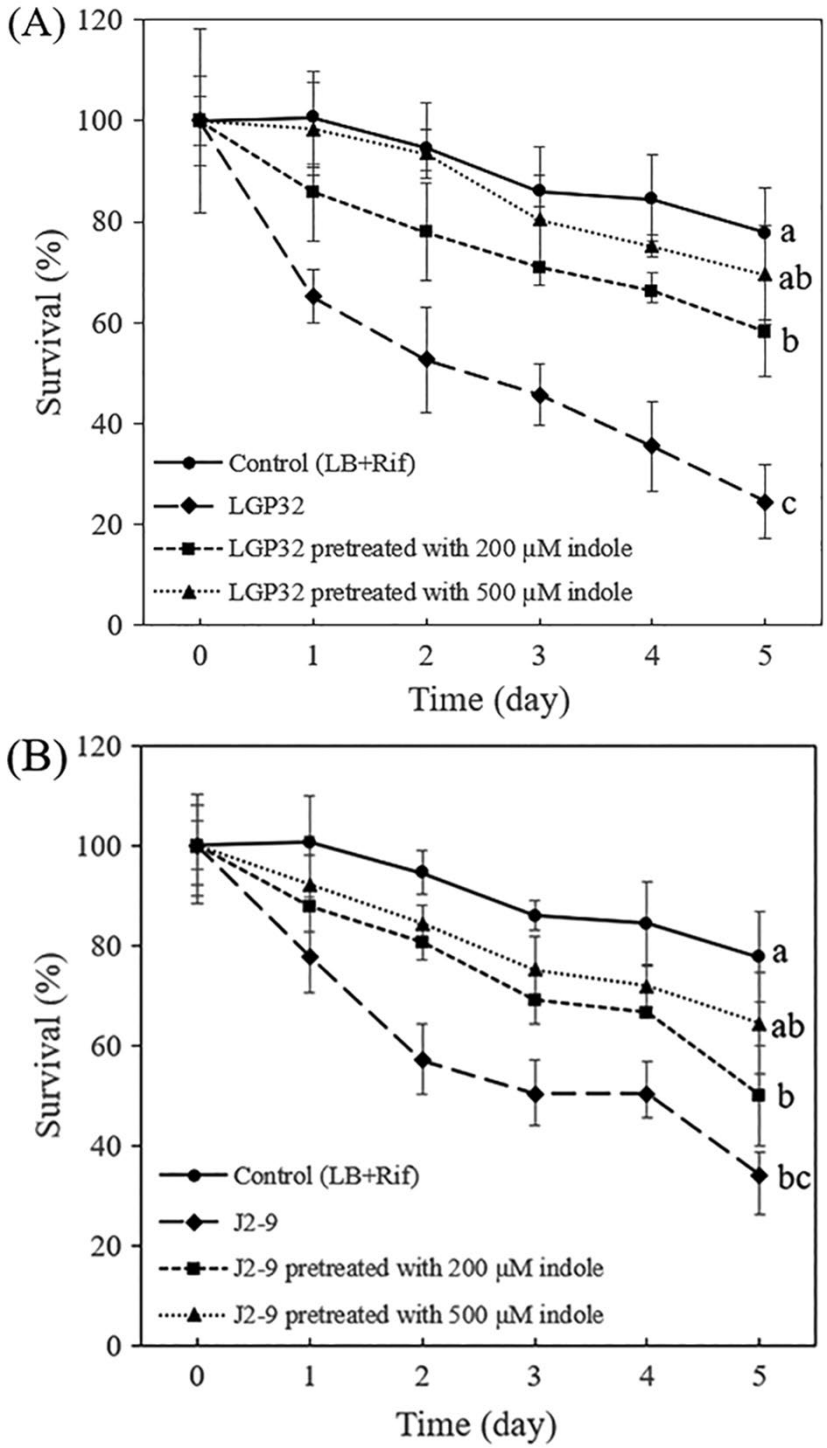
Percent survival of mussel larvae after 1, 2, 3, 4 and 5 days of challenge with *V. tasmaniensis* LGP32 (**A**) and *V. crassostreae* J2-9 (**B**), either or not pretreated with 200 or 500 µM indole. Error bars represent the standard deviation of 4 replicate mussel cultures. Different letters indicate significant differences at the last time point (One way ANOVA with Tukey’s post hoc test; P < 0.01).

To explore the impact of indole on the virulence of *V. tasmaniensis* LGP32 and *V. crassostreae* J2-9 towards mussel larvae, vibrios pretreated with different concentrations of indole were added to mussel larvae. The pathogens were pretreated with indole, and washed before addition to the mussel larvae in order to exclude any direct effect of indole on the larvae. Both *V. tasmaniensis* LGP32 and *V. crassostreae* J2-9 showed to be pathogenic to mussel larvae, leading to significant mortality after 5 days of challenge (Fig. 6). However, the treatment with indole improved the survival of the larvae, especially at the higher concentration of indole. Compared to the treatment without indole, 200 µM indole improved the larvae survival by 2.4-fold, while 500 µM indole improved it by 2.8-fold in *V. tasmaniensis* LGP32 (Fig. 6A). In *V. crassostreae* J2-9, 200 and 500 µM indole increased the larvae survival by 1.5-fold and 1.9-fold, respectively (Fig. 6B).

## Discussion

In the present study, we investigated the impact of indole on virulence factor production and virulence of *V. tasmaniensis* LGP32 and *V. crassostreae* J2-9 towards mussel larvae. We found that pretreatment of *V. tasmaniensis* LGP32 and *V. crassostreae* J2-9 with indole before inoculation into the mussel larvae rearing water resulted in decreased mortality when compared to the larvae that were challenged with untreated pathogens. The higher the concentration of indole, the lower the mortality of mussel larvae was. This indicated that elevated indole levels significantly decreased the virulence of *V. tasmaniensis* LGP32 and *V. crassostreae* J2-9. The results obtained in this study are consistent with our previous observation that indole decreases the virulence of *Vibrio campbellii*, *V. parahaemolyticus,* and *V. harveyi* strains towards gnotobiotic brine shrimp (*Artemia franciscana*) larvae^27,28^, and that of *V. anguillarum* NB10 towards sea bass (*Dicentrarchus labrax*) larvae^26^. All these data further indicated that indole signaling has the potential to be an effective target for antivirulence therapy in aquaculture. However, for practical applications we will need to find indole analogues that do not affect the cultured organisms because adding indole to the rearing water affects fish larvae (positive effect^26^), brine shrimp larvae (negative effect^27^) and mussel larvae (negative effect^29^).

Indole displays a diverse range of effects on bacterial physiology and metabolism^27,30^, yet its molecular targets and mechanisms of action remain obscure^24^. To determine the mechanisms by which indole reduces the virulence of *V. tasmaniensis* LGP32 and *V. crassostreae* J2-9, we evaluated the impact of indole on biofilm formation, swimming and swarming motility. Swimming motility (in liquids) and swarming motility (on wet surfaces) are common modes of motility, both are important throughout the bacterial infection^31^. Swimming motility enables bacteria to detect and pursue nutrients, and move toward environments of favorable conditions^32,33^. Swarming motility allows bacteria to rapidly colonize a surface, leading to the formation of biofilms^34^. Indole decreased swimming and swarming motility in both *V. tasmaniensis* LGP32 and *V. crassostreae* J2-9. These effects were confirmed at the transcriptional level by transcriptomic analysis and reverse transcriptase qPCR targeting key genes involved in these phenotypes. The expression of genes related to bacterial flagella and chemotaxis was repressed by indole. This result is similar to that obtained for *Salmonella enterica* serovar Typhimurium^35^ and *V. campbelli*^27^ in which indole repressed motility and the production of flagella. Further, indole also decreased the biofilm formation of *V. crassostreae* J2-9 but did not affect the biofilm formation of *V. tasmaniensis* LGP32. Indeed, the direction of the effect (up- or downregulation of biofilm formation) caused by indole is different in different bacterial species. For example, indole inhibited biofilm formation in *Acinetobacter oleivorans*^36^, *Bdellovibrio bacteriovorus*^37^, *Pseudomonas aeruginosa*^38^, *E. coli*^39^, *S. enterica* serovar Typhimurium^35^ and *V. campbellii*^27^, while it increased the biofilm formation in *Agrobacterium tumefaciens*^40^ and *Burkholderia unamae*^41^. Additionally, indole had no impact on the motility of *V. anguillarum* in our previous study^26^. These differences might reflect differences in the life styles of the bacteria.

According to the transcriptomic analysis, indole treatment elevated the expression of ATP synthase and genes involved in the ABC transporter permease in LGP32. Likewise, Kim et al.^42^ conducted a transcriptome analysis for *Pseudomonas putida* KT2440 under indole treatment. They demonstrated that 47 genes were differentially expressed, of which 12 genes involved in chaperone and protease functions. Subsequent biochemical analyses showed that the presence of indole improved the membrane perturbation and promoted higher expression of genes associated with TCA cycle, resulting in the decrease of the ATP concentration inside cells. Our results suggest that a similar mechanism might be present in *V. tasmaniensis* LGP32.

Indole is a signaling molecule produced by many bacterial species and involved in intraspecies, interspecies, and interkingdom signaling^24^. To date, more than 85 bacterial species have been found to produce indole^23^. In this study, the concentration of indole produced reached about 200 µM and 250 µM in *V. tasmaniensis* LGP32 and *V. crassostreae* J2-9, respectively. It is well recognized that the accumulation of extracellular indole can be affected by environmental factors, such as the cell density, carbon sources, temperature and pH^43^. In this study, the levels of indole produced by *V. tasmaniensis* LGP32 and *V. crassostreae* J2-9 were dependent on cell density, which is similar to what has been reported for *Edwardsiella tarda*^44^, *E. coli*^45^, *V. anguillarum*^26^ and *V. campbellii*^27^.

In conclusion, indole showed protection of blue mussel larvae against *V. tasmaniensis* LGP32 and *V. crassostreae* J2-9 without affecting bacterial growth*.* This is consistent with the concept of antivirulence therapy, which does not kill the pathogens^19^. Further, indole was found to control different phenotypes in *V. tasmaniensis* LGP32 and *V. crassostreae* J2-9, including biofilm levels, swimming and swarming motility and mRNA levels of genes responsible for these phenotypes. All of the results indicate that indole signaling has the potential to be used as a target for antivirulence therapy in blue mussel larviculture. Interestingly, when compared to conventional antibiotics, interfering with signaling mechanisms (like indole signaling) is expected to impose less selective pressure (no impact on growth) on bacteria to evolve resistance leading to a lower chance of resistance development (although it cannot be excluded at this moment). Indeed, we did not observe any trend towards resistance in our study, nor has resistance to indole been documented for any other bacterium.

## Methods

### Bacterial strains, culture conditions, and chemicals

*Vibrio tasmaniensis* LGP32 and *V. crassostreae* J2-9 were cultured in Luria–Bertani medium containing 35 g/L of sodium chloride (LB_35_) at 28 °C under constant agitation (100 min^−1^). Cell densities were measured spectrophotometrically at 600 nm. Indole, purchased from Sigma-Aldrich (Belgium), was dissolved in methanol at 100, 200, 500 mM, respectively. In all experiments, all treatments received the same volume of methanol.

### Quantification of indole

*Vibrio tasmaniensis* LGP32 and *V. crassostreae* J2-9 cultures were grown overnight in LB_35_ broth (reaching OD_600_ of 1) and re-inoculated (1% v/v) into fresh LB_35_ broth. Cell free supernatants from the cultures were obtained at the time intervals of 6, 12, 24 and 48 h by centrifugation at 8000×*g*, followed by filtration through 0.22 µm membrane filters. The concentration of indole in the supernatants was measured as described previously^26^ by mixing 500 µL of supernatant with 500 µL of Kovac’s reagent. After vortexing, the top 200 µL was removed and the OD_571_ was measured. The indole concentration in each sample was determined based on a standard curve using synthetic indole (Sigma-Aldrich). At least three different cultures were sampled for each strain at each time point.

### Impact of indole on bacterial growth

To investigate the effect of indole on the growth of *V. tasmaniensis* LGP32 and *V. crassostreae* J2-9, overnight grown cells were inoculated into fresh LB_35_ media at an initial OD_600_ of 0.01. Indole was added at 0, 100, 200 and 500 µM, respectively. Then 200 µL aliquots of these suspensions were pipetted into the wells of a polystyrene 96-well plate and cultured in Tecan Infinate M200Pro plate reader at 28 °C for 40 h. The OD_600_ of each sample was measured by a Tecan Infinate M200Pro plate reader every hour. Growth curves were determined for three independent cultures.

### Swimming and swarming motility assays

The swimming motility assay was determined on LB_35_ soft agar plates containing 0.2% agar^46^, and the swarming motility assay was determined on LB_35_ swarming agar plates containing 0.8% agar^47^. *Vibrio tasmaniensis* LGP32 and *V. crassostreae* J2-9 were grown overnight in LB_35_ medium, and diluted to OD_600_ of 1. The LB_35_ soft agar was cooled down to approximately 50 °C after autoclaving, then indole was added at concentrations of 0, 200 and 500 µM, respectively. The agar was poured into petri plates and left open at room temperature for 15 min. Five µL aliquots of the bacterial suspensions were added to the center of soft agar plates (6 replicate plates per treatment). The plates were incubated upright at 28 °C and the motility halo diameters were measured every day.

### Biofilm formation

Biofilm formation was quantified by crystal violet staining, as described previously^48^. Briefly, overnight cultures of *V. tasmaniensis* LGP32 and *V. crassostreae* J2-9 were diluted to OD_600_ of 0.1, and indole was added at concentrations of 0, 200 and 500 µM, respectively. Then 200 µl aliquots of these suspensions were pipetted into the wells of a polystyrene 96-well plate and cultured without agitation at 28 °C for 24 h. After that, unattached cells were washed away with PBS for three times. Then the remaining attached bacteria were fixed with 200 µL methanol per well for 20 min, after which the methanol was removed and the plates were air-dried. Then, biofilms were stained for 15 min with 200 µL per well of a 1% crystal violet solution. Plates were then rinsed with running water until the washings were free of the stain. After the plates were air-dried, bound crystal violet was dissolved in 200 µL of 95% ethanol per well for 30 min, and absorbance was measured at 570 nm with a Tecan Infinate M200Pro plate reader. Sterile medium served as negative control, and the reported values are blank-corrected.

### Genome-wide transcriptomic analysis of the impact of indole on *V. tasmaniensis* LGP32

*Vibrio tasmaniensis* LGP32 was grown in LB_35_ medium supplemented with or without indole at 500 µM with shaking at 28 °C for 6 h. The total RNA was extracted using the RNeasy Protect Bacteria Mini Kit (Qiagen, Hilden, Germany) as per the manufacturer’s recommendation, and paired-end sequencing was performed on the Illumina Hi-Seq 2000 platform of the LC-Bio Technology Co., Ltd. (Hangzhou, Zhejiang, China). The raw transcriptomic sequencing data were submitted to GenBank (NCBI) under the BioProject No. PRJNA741367. Raw reads of transcriptome sequencing were mapped to genome of LGP32 by Bowtie2-2.2.3. Differential expression analysis was performed for three biological replicates using the DESeq R package (1.18.0). Genes were considered differentially expressed with over 1.5-fold change and p-values < 0.01.

### RNA extraction and quantitative reverse transcription PCR (RT-qPCR)

Overnight cultured *V. tasmaniensis* LGP32 and *V. crassostreae* J2-9 were diluted to 1% in fresh LB_35_ medium supplemented with or without indole at 200 and 500 µM. Each sample was grown in triplicate at 28 °C for 6 h. RNA extraction and reverse transcriptase real-time PCR were performed as described previously^46^. Total RNA was extracted using the SV Total RNA Isolation System (Promega) and DNA contamination was eliminated with a DNase treatment (Thermo Scientific Rapid Out DNA Removal Kit). The RNA quantity was measured spectrophotometrically (NanoDrop Technologies) and adjusted to 200 ng/µL in all samples. The RNA quality was confirmed with Agarose Gel Electrophoresis and the RNA samples were stored at − 80 °C. The cDNA was synthesized from RNA with reverse transcriptase using the ProtoScript (r) II First Strand cDNA Synthesis Kit (New England biolabs) according to the manufacturer’s instructions. Briefly, 1 µg RNA, 2 µL random primer and Nuclease-free H_2_O was mixed to a total volume of 8 µL. Then the samples were denatured for 5 min at 65 °C and put promptly on ice. After that, 10 μL of 2 × Reaction Mix and 2 μL of 10 × Enzyme Mix were added. The 20 μL cDNA synthesis reactions were incubated at 25 °C for 5 min, followed by 60 min at 42 °C and 80 °C for 5 min. Finally, cDNA samples were cooled to 4 °C, were checked by PCR and stored at − 20 °C for further use.

Real-time qPCR was carried out in a StepOne ™ Real-Time PCR System thermal cycler (Applied Biosystems, Gent, Belgium) by using the Luna Universal qPCR Master Mix (New England biolabs). The primers for qPCR are listed in Table 2, and each assay was performed in triplicate, expression of 16S rRNA was used as the internal reference gene to normalize the expression of the genes in Table 2, with the primers of 933F and16SRTR1. The comparative threshold cycle method (2^−ΔΔCT^)^49^ was used to analyze the relative mRNA level.

**Table 2:** Primers used in this study.

Gene	Function	Primer sequences (5′–3′)
**Primers for *Vibrio tasmaniensis* LGP32**		
*flaG*	Flagellar protein FlaG	F: CAAGGGTGTTGCTTTTAAGG
		R: ACTATTCGAGGTTTGGGCTG
*flaN*	Flagellin	F: CTAAACGGTCAAAGCCCAGA
		R: CAAGAGAGCCGCCAAACTCA
*flaB*	Flagellin B	F: ATTACTGCCTCACCATT
		R: TCGCTGAAACAACCTCT
*pflaB*	Polar flagellin B	F: TGGGGAAATTGCATTTTCTG
		R: AGCCTCTAGCGTTTTTGGGT
*tnaA*	Tryptophanase	F: TGCAAGAAGGCTTCCCAAC
		R: ATGAACCTGACCAATACGG
*cspA*	Carbon starvation protein A	F: CGCCATCAAAGGAGACT
		R: ACCAACATGAACCCGAA
*tcS*	Two-component system response regulator	F: GCACTTTATGACCAACAA
		R: AAATTCAGCAGCTCTACC
*ABC tpr*	ABC transporter permease-related gene	F: CTGTGGCAGGCTTTGGT
		R: CGTGAGTGGCGTTTCTT
*atpE*	Putative ATP synthase subunit C	F: CAGACCTTACTCCAATG
		R: TAGACCGATACCAACAC
*933F*	Internal reference gene	F: GCACAAGCGGTGGAGCATGTGG
*16SRTR1*		R: CGTGTGTAGCCCTGGTCGTA
**Primers for *Vibrio crassostreae*** **J2-9**		
*fliF*	Flagellar M-ring protein FliF	F: GTTGATATTCAGCTCGA
		R: GCCACAGTATTACCGTT
*flaG*	Flagellar protein FlaG	F: GGTAGGGATGTGGTGAC
		R: ATTTGAGTTTTGGGCTG
*flgE*	Flagellar hook protein FlgE	F: TCAGTTTGCTGCTCCGTTT
		R: CCATTGAGTGCCACCTTTC
*cspA*	Carbon starvation protein A	F: CCCGAATGACCTACCACTT
		R: TCTCGTTTTCCATACAACG
*pmbA*	Metalloprotease PmbA	F: AACGGTGAAATGGAACG
		R: CAAACGGCTAATGGTCT
*933F*	Internal reference gene	F: GCACAAGCGGTGGAGCATGTGG
*16SRTR1*		R: CGTGTGTAGCCCTGGTCGTA

### Challenge tests with blue mussel D-larvae

Challenge tests were performed as described previously^7^. Wild-caught mature blue mussels were stimulated to spawn by thermal shocks in autoclaved seawater at 5 °C and 20 °C until gametes were released. Spawning males and females were transferred to sterile plastic cups containing 50 mL sterile seawater and allowed to spawn for 15 min. Sperm and eggs were collected and gently mixed at a 10:1 ratio in a beaker containing 1 L of sterile seawater. After the appearance of polar bodies, the eggs were gently rinsed with sterile seawater using a sterile 30 μm sieve to remove excess sperm. Fertilized eggs were incubated in 2 L of sterile seawater (max 100 eggs/mL) containing chloramphenicol, nitrofurazone and enrofloxacin (each at 10 mg/L). After two days of incubation, D-larvae were harvested on a sterile 60 μm sieve. The larvae were washed gently with sterile seawater to remove the antibiotics. Rinsed D-larvae were transferred to a beaker containing 1 L of sterile seawater and distributed uniformly using a plunger. Subsamples were taken to calculate the larval density, and the density was corrected to obtain a final concentration of 250 larvae/mL. All manipulations were performed under a laminar flow hood.

Natural rifampicin-resistant mutants *V. tasmaniensis* LGP32 and *V. crassostreae* J2-9 were cultured overnight and transferred to fresh LB_35_, and further cultured with 200 µM and 500 µM indole for 6 h, respectively. The treatment without indole contained the same volume of methanol. One mL aliquots of the larval suspension in seawater supplemented with 10 mg/L tryptone and 5 mg/L yeast extract were subsequently transferred to 24-well plates. Rifampicin was added at 10 mg/L to avoid contamination. The final larval density was 200 larvae/mL. Natural rifampicin-resistant mutants of *V. tasmaniensis* LGP32 and *V. crassostreae* were inoculated into the rearing water at 10^5^ cells/mL. Larvae to which no bacteria were added and that were otherwise treated in the same way as challenged larvae, were used as controls. Each treatment was performed in 24 replicates. The plates were incubated at 18 °C. Each day, four replicates per treatment were stained with lugol [5% (v/v)], and stained larvae were counted under a binocular microscope (Nikon Eclipse E 200, Nikon Instruments Europe). Larvae were considered alive when stained black by lugol, death if only parts of the larvae were stained or if shells were empty.

## Supplementary Information

Supplementary Table S1.

## Data Availability

The raw transcriptomic sequencing data were submitted to GenBank (NCBI) under the BioProject No. PRJNA741367. The other datasets generated during and/or analysed during the study are available from the corresponding author upon request.
